# Reach and perceived effectiveness of a community-led active outreach postvention intervention for people bereaved by suicide

**DOI:** 10.3389/fpubh.2022.1040323

**Published:** 2022-12-22

**Authors:** Nicole T. M. Hill, Roz Walker, Karl Andriessen, Hamza Bouras, Shawn R. Tan, Punam Amaratia, Alix Woolard, Penelope Strauss, Yael Perry, Ashleigh Lin

**Affiliations:** ^1^Telethon Kids Institute, Nedlands, WA, Australia; ^2^Centre for Child Health Research, University of Western Australia, Nedlands, WA, Australia; ^3^School of Population and Global Health, University of Western Australia, Nedlands, WA, Australia; ^4^Ngangk Yira Institute for Change, Murdoch University, Murdoch, WA, Australia; ^5^School of Indigenous Studies, University of Western Australia, Perth, WA, Australia; ^6^Melbourne School of Population and Global Health, University of Melbourne, Carlton, VIC, Australia

**Keywords:** postvention, suicide prevention, suicide cluster, community intervention, suicide prevention and intervention

## Abstract

**Background:**

Postvention is a core component of suicide prevention strategies, internationally. However, the types of supports provided to people impacted by suicide vary widely. This study examines the perceived effectiveness of the Primary Care Navigator (PCN) model for people bereaved by suicide. The PCN model was implemented in response to a suicide cluster. It is an active outreach postvention intervention, initiated by police in response to a suspected suicide and links individuals to support in the immediate aftermath of their loss.

**Methods:**

A retrospective cross-sectional mixed methods approach was used to (1) identify the reach of the PCN model, (2) describe the type of support provided to people bereaved by a suspected suicide and (3) identify the perceived effectiveness of the PCN model from the perspective of WA police, postvention stakeholders and individuals bereaved by suicide. Quantitative data was used to examine the characteristics of suicide in the region, the characteristics of people who received bereavement support, and the types of support that were provided. Interviews with police, postvention stakeholders, and people bereaved by a suspected suicide were conducted to identify the perceived effectiveness of the intervention.

**Results:**

Between 1 January 2019 and 31 March 2021 there were 80 suspected suicides. Active outreach was provided to 347 bereaved individuals via the PCN model. Just under half of those who were offered outreach accepted further support (*N* = 164) in the form of suicide bereavement information (98%), mental health or clinical support (49.6%), specialized postvention counseling (38.4%), financial assistance (16%) and assistance with meals (16%), followed by housing assistance (14%) and referral to community services (11%). Police, stakeholders, and people with lived experience of a suspected suicide perceived the PCN model to be effective at connecting them to the community, linking people to support, and preventing suicide.

**Conclusion:**

The results provide evidence supporting the perceived effectiveness of an active outreach approach to postvention that provides acute support to people bereaved by suicide. Findings highlight important practical areas of support such as providing referral pathways and information on grief and suicide loss in the immediate aftermath of a suicide loss.

## Introduction

Suicide prevention is a significant public health priority in Australia. In 2020, 3,139 Australians died by suicide representing a rise from 11.2 per 100,000 in 2019 to 12.1 per 100,000 in 2020 (1). It is estimated that for every suicide approximately five or more immediate family members are affected and up to 135 individuals within the broader community (2, 3). A recent meta-analysis showed that approximately 1 in 20 people are impacted by a suicide in the past year, whereas 1 in 5 individuals will be impacted by a suicide during their lifetime (4).

Suicide bereavement is associated with an increased risk of adverse physical and mental health outcomes (5, 6), including increased risk of suicide, suicide attempt (7, 8), and the development of suicide clusters (multiple suicides that occur close in space and time, or those that involve social links between cluster members) (9). Access to timely postvention, defined as activities that provide support and facilitate recovery in those bereaved by suicide (10), has been identified as a core component of local, state-and national suicide prevention strategies in Australia and internationally (11, 12). Furthermore, timely postvention is considered a gold-standard approach for the prevention of suicide clusters (13).

To date, most literature has examined the effectiveness of psychological interventions (e.g., bereavement counseling) on people bereaved by suicide (14, 15). Evidence from controlled studies, for example, suggest that engagement in psychological interventions following a suicide loss is associated with some improvements in grief and suicidal ideation compared to those who do not receive psychological support (14). However, the needs of people bereaved by suicide varies widely between individuals and at different stages post-loss (16–18). A recent qualitative study by Ross and colleagues, for example, found that in addition to psychological needs, individuals bereaved by suicide experience a range of practical needs including assistance with funeral arrangements, managing finances and assistance accessing appropriate psychological support to guide them through the bereavement process (17). Moreover, the type of support that is needed and sought may depend on factors such as the availability of social support and relationship to the deceased. For example, Entilli et al. found that individuals with high social support were less likely to seek support from formal services (e.g., psychiatric services) compared to those with low social support (19). Another study found people with different family roles may require different types of support, with mothers reporting more frequent symptoms of depression compared to fathers (18).

Furthermore, previous research shows, many people bereaved by suicide would like to receive support following their loss but for various reasons are not able to access the help they need (20–23). Barriers include long waitlist times (or the absence of services altogether), lack of clarity regarding where to look for support, or the belief that service providers may not think their problems are serious enough to warrant support (21, 23). Arguably, postvention interventions which address these barriers in the immediate aftermath of a suicide have significant potential for improving access to postvention support for individuals impacted by suicide.

There is some evidence that those who receive practical postvention support in the immediate aftermath of a suicide are more likely to engage in interventions targeting their psychological recovery. Cerel and Campbell found that bereaved individuals who received practical support in the immediate aftermath of their loss presented to postvention counseling services on average 50 days sooner than those who did not receive practical support soon after their loss (24). The type of postvention outreach provided to people in the immediate aftermath of a suicide has also been linked to improved outcomes in people bereaved by suicide. For example, previous studies have shown that people who receive active postvention outreach (where outreach and support is initiated by a support service or organization) as opposed to passive postvention (where individuals are provided with passive information about supports and/or are required to initiate support themselves) is associated with better psychosocial outcomes including fewer work-related absences, less contact with health professionals, and improved engagement in psychological supports (16, 24, 25). Despite the potential benefits, active outreach is infrequently provided. For example, in a survey of people bereaved by suicide, 18 found less than 15% of people had received direct outreach during the aftermath of their loss. Yet, 90% of those who received outreach were happy with the support they were provided (19).

Despite promising evidence, the acceptability and perceived effectiveness of active postvention outreach remains limited. It is not currently known whether an active outreach model is feasible from a service delivery perspective, or whether bereaved individuals who receive active outreach perceive the approach as beneficial for their recovery. The aim of the present study is to address these gaps in evidence in a descriptive study that investigates the reach and perceived effectiveness of an active outreach model known as the Primary Care Navigator (PCN) model implemented in the PaRK region in Western Australia (WA). Specifically, we sought to: (1) identify the reach of the Primary Care Navigator model, (2) describe the type of support provided to people bereaved by a suspected in the region, and (3) identify the perceived effectiveness of the PCN model from the perspective of WA police, postvention stakeholders and individuals bereaved by suicide. In the current study we refer to the deaths as suspected suicides as not all had been confirmed by the coroner, which can take up to 2 years following a sudden death notification from police.

## Methods

### Local context and intervention description

In 2016–2017 a cluster of suicides occurred in southwest metropolitan Perth (PaRK region). In response to the deaths, stakeholders from local mental health services, WA Primary Health Alliance, and WA police developed the Primary Care Navigator Model. The purpose of the PCN model is to link individuals to practical postvention support in the form of active outreach in the immediate aftermath of a suspected suicide.

In Australia, the loss of an individual to a suspected suicide requires the involvement of the state coroner to investigate both the cause and circumstances of death (26). This process is facilitated by police, who work on behalf of the coroner and are required to identify the individual who died, collect statements from caregivers, close contacts, and witnesses, and notify individuals and families that a death has occurred. Whilst the purpose of police is to assist with the collection of information on behalf of the coroner, they are often the first to contact individuals bereaved by suicide.

**Figure 1:**
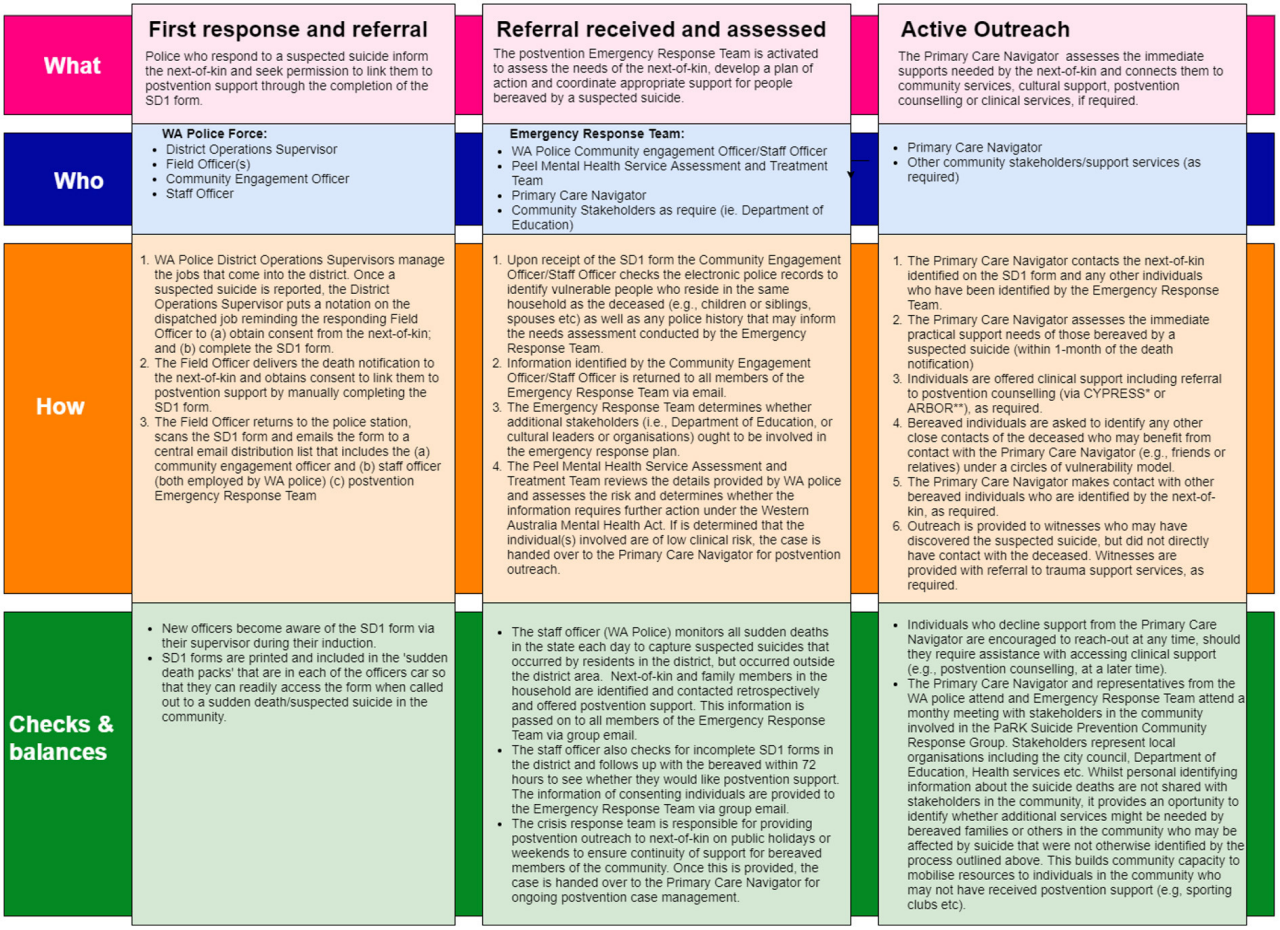
Operational description of the primary care navigator (PCN) model.

Under the PCN model, police ask bereaved individuals for their consent to receive active outreach via a sudden death notification form (SD1 form), which is facilitated by the PCN lead. The SD1 form records the demographic characteristics and other individuals nominated by the bereaved person who may benefit from postvention outreach. The rapid referral between police and the PCN lead was designed to provide immediate postvention support to those who are bereaved by suicide with the aim of improving recovery and preventing further suicides and suicide attempts. A suicide prevention community response group comprising stakeholders from community organizations (e.g., local council, mental health services, and schools) meet monthly to identify opportunities for suicide prevention. When a suicide occurs, the response group reviews deidentified information on the deceased (e.g., demographic information) to identify other individuals in the community who may benefit from outreach and support (e.g., young people in community sports groups). A summary of PCN model is shown in Figure 1.

The study used a cross-sectional mixed methods approach. We analyzed quantitative data that described the characteristics of individuals who died by suspected suicide and individuals bereaved by suspected suicide who received active postvention outreach from the PCN model between 1 January 2019 and 31 March 2021 in the PaRK region in Western Australia. We obtained data on suspected suicides from the SD1 forms, recorded by the service provider (Anglicare WA) responsible for providing bereaved individuals with active postvention support under the PCN model. We collected qualitative data from semi-structured interviews with people bereaved by suicide, stakeholders and police involved in the delivery of the PCN model.

### Quantitative analysis

#### Data sources

##### Ascertainment of suspected suicide deaths and bereaved participants

We identified suspected suicides that occurred between 1 January 2019 and 31 March 2021 from a spreadsheet collected by the Primary Care Navigator for all people who died by suspected suicide and those who received active outreach. Each bereaved individual was linked to the case record of a suspected suicide using a numerical code generated by the service provider (Anglicare WA). A list of the variables provided by Anglicare WA to the research team for descriptive analysis is shown in Table 1.

**Table 1:** Variables included in the quantitative analysis.

**Variable**	**Description**
Date of police referral to the PCN	Day, Month, Year.
Case number	A unique number linked to the deceased person.
Relationship to the deceased	Describes the relationship to the person who died by suicide (e.g., First degree relative – spouse, sibling, parent, stepparent, step siblings, ex-spouse, other relative; OR Acquaintance: neighbor, employee, colleague, friend, other).
Gender	Describes the persons gender identity (e.g., Male, Female, Nonbinary, Trans male Trans Female).
Age (numerical number)	Age at the time of contact with the PCN
Postcode	Residential postcode.
The number of referrals to community services that the PCN provided to the client.	Numerical descriptor.
The types of services included in each referral	The type of referrals that were made (e.g., clinical, funeral, financial, biological hazard clean-up, postvention counseling, mental health services).
The number of calls/contacts made per client	Total number per client.
Date of suspected suicide	
Gender of the deceased person	Describes the persons gender identity (e.g., Male, Female, Nonbinary, Transgender).
Age of the deceased person	Age at the time of police notification.
Postcode of the deceased person	As above.
Method of suspected suicide	The mechanism resulting in death.

##### Ascertainment of population size

We used the Australian Bureau of Statistics (ABS) Table builder to extract data on estimated resident population for 2019 (the midpoint of our study) for each of the five local government areas included in the catchment region. Age was coded into 5-year bands from ages 15-85+ to reflect the age of the population who died by suspected suicide in the PaRK region.

##### Statistical analysis (quantitative data)

Age specific rates of suspected suicide per 100,000 person years were calculated for each age group. We used descriptive statistics to summarize the characteristics (by gender) of those who died by suspected suicide as well as bereaved people who received outreach from the PCN lead. Differences between sexes was determined using Chi-square analysis with significance determined at the p < 0.05 level. The reach of the PCN service was analyzed using descriptive statistics that summarized the ratio of outreach contacts made per suspected suicide in addition to the frequency and type of outreach provided to individuals who received outreach through the PCN model during the study period. All quantitative analysis was conducted in R v3.8.

### Semi-structured qualitative interviews

Semi structured qualitative interviews were conducted online via Microsoft Teams with 5 bereaved individuals, 18 stakeholders from the PaRK Suicide Prevention Response Group (SRG) and 5 employees of the WA police force who were involved in the implementation and delivery of the PCN model. Key topics covered in the semi structured interviews included: 1) the operational efficiency of the PCN model; 2) the strengths and barriers associated with the model; and 3) the perceived effectiveness of the model for people bereaved by suicide in the community. The current study focuses on responses to the perceived effectiveness of the PCN model and areas for future improvement.

#### Recruitment

All participants were recruited between 1 July 2021 and 1 October 2021. A purposive sampling method was employed for each participant group. Bereaved participants were recruited by the PCN lead via email, and stakeholders were recruited by the Suicide Prevention Project Support officer using the email distribution list for the local suicide response group, hosted by Anglicare WA. WA Police were recruited by the research team using an email distribution list of police who have responded to a suspected suicide. The email distribution list was prepared by the district Inspector of police in the region.

#### Qualitative analysis

Two researchers (RW and NTMH) conducted the interviews between 1 September 2021 and 31 October 2021. Convergent themes between groups (e.g., stakeholders, police, and people with lived experience) were identified using a general inductive approach (27). Emerging themes were defined as experiences or opinions that shared similar content or meaning. A theme was identified as a convergent theme if it was reported across all three participant groups (stakeholders, police and lived experience). RW and NTMH each generated emerging themes and discussed the results until consensus was reached. To facilitate deep engagement with the data, NTMH checked the recordings against the transcriptions for accuracy. Analysis of the data was iterative and involved: familiarization with the data; generating themes; coding; reviewing themes; naming and defining themes; and synthesizing the results in the written manuscript (28–30). Segments of interest that provided meaningful insight into the semi-structured interview questions were identified and coded (28). The coding process was flexible and iterative and emerging themes were revised through discussion between RW and NTMH (29, 30).

Recordings were transcribed by a professional transcription service. Transcripts were imported into QSR NVivo 11 to facilitate data management and analysis. Two interviews were not successfully recorded and were therefore included on the basis of field notes collected by the interviewer.

### Ethics approval

The study was approved by the University of Western Australia Human Research Ethics Committee (2021/ET0000306) and the Western Australia Police Force Research Governance Office (T570). All participants provided written informed consent.

## Results

### Results of the quantitative analysis

#### Characteristics of suspected suicide in the region (2019–2021)

Between 1 January 2019 and 31 March 2021 there were 80 suspected suicides that were referred by police to the PCN via the SD1 referral form (Table 2). Males accounted for 80% of suicides during the study period. The overall age specific rate of suspected suicide in the PaRK region between 2019 and 2021 was 26.2 per 100,000 people. The age specific rate of suspected suicide was highest among young people aged 20–24 years (65.9 per 100,000 people), followed by those aged 15–19 years (45.1 per 100,000 people). Together, young people aged 15–24 accounted for 35% of suspected suicides within the PaRK region during this period. Most people who died by suspected suicide in the region were adults over the age of 18 (90%; Table 2). Over half were known to inpatient and outpatient alcohol and other drug services (53.8%) and/or community mental health services (50.8%). Overall, 11.3% of those who died by suicide were exposed to the suicide of a relative, friend or acquaintance during their lifetime.

**Table 2:** Characteristics of those who died by suspected suicide in the PaRK region, 2019 to 2021.

**Characteristics**	**Total**	**Male**	**Female**	**Chi square *p*-value**
Number of people	80 (100%)	64 (80.0%)	16 (20%)	<0.01
Suspected suicides in 2019	17 (21.3%)	14 (82.4%)	3 (17.6%)	<0.01
Suspected suicides in 2020	46 (57.5%)	35 (76%)	11 (24%)	<0.01
Suspected suicides in 2021	14 (17.5%)	13 (92.9%)	1 (7.1%)	<0.01
15 to 19	8 (10.0%)	7 (10.9%)	1 (6.7%)	0.61
20 to 24	12 (15.0%)	6 (9.4%)	6 (40.0%)	<0.01
25 to 29	8 (10.0%)	7 (10.9%)	1 (6.7%)	0.61
>30 years	52 (65.0%)	43 (67.2%)	9 (60.0%)	0.58
Aboriginal and Torres Strait Islander	9 (11.3%)	8 (12.5%)	1 (6.7%)	0.52
Exposed to suicide (lifetime)	10 (12.5%)	8 (12.5%)	2 (13.3%)	0.93
Known to Community Mental Health Services	47 (58.8%)	34 (53.1%)	13 (86.7%)	0.01
Known to Alcohol and Other Drug Services	43 (53.8%)	37 (57.1%)	6 (40.0%)	0.22

#### Characteristics of individuals bereaved by suicide

The characteristics of individuals bereaved by suicide is shown in Table 3. Females accounted for 63% of bereaved individuals in the community. Of the 80 suspected suicides, postvention outreach in the first 48–72 hours of a suspected suicide loss was provided to 347 bereaved individuals under the PCN model (see Supplementary Figure 1). The number of people who received initial outreach increased from 75 bereaved individuals (20 completed SD1 referrals in response to a suspected suicide) in 2019 to 235 (46 completed SD1 referrals in response to a suspected suicide) in 2020. Approximately 10% of people who were provided outreach from the PCN lived outside the catchment region but were identified by the SD1 form as requiring postvention support.

**Table 3:** Characteristics of individuals bereaved by a suspected suicide, 2019 to 2021.

**Characteristic**	**Total**	**Male**	**Female**	**Chi square**
	**(*N =* 164)**	**(*N =* 60)**	**(*N =* 104)**	***p-*value**
**Age**				
Adult >18 years	73 (44.5%)	40 (60.7%)	33 (31.7%)	<0.01
Minor <18 years	91 (55.5%)	20 (33.4%)	71 (68.3%)	<0.01
**Relationship to the deceased**				
Spouse^*^	25 (15.2%)	2 (3.3%)	23 (22.1%)	<0.01
Parent^*^	29 (17.7%)	10 (16.7%)	19 (18.3%)	0.79
Son/daughter^*^	61 (37.2%)	26 (43.3%)	35 (33.7%)	0.22
Sibling^*^	22 (13.4%)	7 (11.7%)	15 (14.4%)	0.63
Other relative	4 (2.4%)	1 (1.7%)	3 (2.9%)	0.63
First responder or witness	6 (3.7%)	3 (5.0%)	3 (2.9%)	0.49
Friend or acquaintance (colleague, neighbor, housemate)	17 (10.4%)	11 (18.3%)	6 (5.8%)	0.01

* Includes defacto spouse, step-parent(s), step-children, and step-siblings.

#### Reach and nature of support provided by the Primary Care Navigator

On average, postvention outreach was provided to ~4 bereaved persons per suspected suicide (range = 1–16 bereaved individuals). Overall, 164 (47.2%) people accepted postvention support, of which just under half accepted further support in the form of practical support (e.g., financial support, funeral arrangement, etc.) and/or clinical support (e.g., suicide bereavement postvention counseling). Compared to those who accepted support, those who declined further support following initial contact from the PCN were more likely to be male and were more likely to be a friend or acquaintance of the deceased, or a bystander witness who discovered the deceased (Table 3).

#### Types of support received

Table 4 shows the types of support provided to bereaved individuals by the PCN. Of the 164 of bereaved individuals who accepted outreach by the PCN, 92.7% received a referral for clinical or specialized postvention counseling, of which more than three-quarters attended at least one appointment.

**Table 4:** Description and frequency of support provided to bereaved people in the PaRK region.

**Practical support**	**Examples of support provided**	**Proportion of bereaved people who received support *n*(%)^a^**
Mental health/clinical services	Child and Adolescent Mental Health Services Private counseling services	81 (49.6%)
Specialized postvention counseling	Children and Young People Responsive Suicide Support (CYPRESS) Active Response Bereavement Outreach (ARBOR)	63 (38.4%)
Suicide bereavement information	Information on how to talk with children about suicide and the suicide death of their loved one Information on suicide related bereavement and grief processes	160 (98%)
Financial support	Vouchers for household items and groceries Assistance finding support for funeral costs Information on services to assist with legal costs Assistance with managing general bills and utilities Assistance with rent or mortgage support	26 (16%)
Meals	Identifying support networks to assist with meals.	26 (16%)
Housing assistance	Assistance to navigating the potential loss of home due to inability to pay rent or lease discontinuation Connecting families to housing support	23 (14%)
Referral to community services	Headspace Youth services Community services Indigenous postvention services Indigenous community development services	18 (11%)
Legal services	Closing of personal affairs/accounts Rent/housing advocacy following the loss of a primary income earner	7 (<5%)
Funeral support	Locating suitable premises fo*r* funeral Assistance with writing a eulogy	9 (5%)
Liaising with Department of Child Protection (DCP)	Collaboration with the Department of Child Protection for at risk families to assess their needs and identify appropriate supports and assess risk and safety for children and young people	7 (<5%)
Transport	Transport assistance to arrange personal affairs in the absence of alternative transport options.	4 (<5%)

##### Results of the qualitative analysis

Four overarching themes were identified in the analysis of perceived effectiveness of the PCN model from participants bereaved by suicide, and stakeholders and police involved in the response to suicide in the region.

##### Linking individuals to the support they need

There was consensus among stakeholders, police and bereaved participants that the PCN model contributed to the coping and recovery of individuals and their family members mental health and grief response. They also described that the support provided by the PCN was needed at the time they received it: “*I had lost my son and he just let me grieve my way but gave me tools to cope I guess. I had lost my brother to suicide seven years prior, and my father had died 6 months before so I had a lot of grief to deal with. They arranged for me to have 20 out of the 10 sessions [bereavement counseling]. They were fantastic, very supportive, they knew I needed more counseling than just ten sessions”* [Bereaved Participant A].

Bereaved participants described receiving valuable information on how to talk to young people in their families about the suicide loss, how to cope with grief through the provision of specialist postvention counseling, that they would not have known about had they not received outreach: “*I really appreciated the fact that that help was offered [by the PCN] because I didn't know it existed”* [Bereaved Participant C].

“*With [my son] then passing, even my brain was telling me, “You need to talk to someone, you do need to talk to someone this time.” So, that's what probably made me go along to the sessions [bereavement counseling arranged by the PCN], and I really didn't know where to start because I've never been and didn't know….By about six, I felt I was – had had enough. I was okay”* [Bereaved Participant B].

One bereaved participant noted that the outreach provided by the PCN helped them process their feelings by talking about what they had experienced and prevented them from “*bottling it all up.”* [Bereaved Participant B]. Another noted: “*I had some really down times and he helped me through those. I had lost my son and he just let me grieve my way but gave me tools to cope”* [Bereaved Participant A].

Both stakeholders and police noted that the model had benefits for their own wellbeing and provided them with the psychological comfort to walk away knowing that the community had an effective strategy in place to support people who received the distressing news of the suicide of a loved one. As one police officer noted: “*it feels like we've [the police] thrown a hand grenade into their household and just let it explode and walk away. Now, when they fill that form [the referral to the PCN], we're not taking that burden home after their night shift because they know that someone is going to pick up and go and help those people”* [Police Participant B].

##### Postvention as suicide prevention

Participants expressed that they thought the support provided by the PCN had the potential to avert further suicides in the community. One police officer noted that the model had the potential to prevent further suicides by providing active outreach when people are most vulnerable “*So, we're trying to break those cycles of the impacts of suicide leading to [more] suicides. So, I think that's incredibly powerful.”* [Police Participant B]. Another stakeholder noted: “*I have no doubt that this initiative has actually saved lives just by – a small thing to us like [the PCN lead] making a phone call to a family can be life-changing to someone who's sitting there on their own”* [Stakeholder Participant A].

The feedback from bereaved participants reinforced those reported by stakeholders. One bereaved participant noted that the support provided by the PCN kept them in close contact with counseling services when they were feeling vulnerable to suicide themselves: “*they knew I needed more counseling than just ten sessions. I mean that is the postvention suicide prevention isn't it, I hit some very low spots, I could have taken my life a few times”* [Bereaved Participant A].

##### Maintaining connection to the community

There was consensus among participants that the PCN model provided people bereaved by suspected suicide with connection in the community by linking them to postvention counseling with other bereaved individuals, and providing a touchpoint for further support, should they need it. One bereaved participant noted: “*Having to go through this experience, it's made it really good to know there's people around you and knowing I can reach out to those people too, that if I do have some concerns for myself or something like that there are people in the background and that's the biggest part that you know you're supported”* [Bereaved Participant C].

Both stakeholders and police echoed the importance of maintaining connection in the community, particularly for individuals who did not have strong support networks at the time of their loss. For example, one police officer noted: “*Some people have their church beliefs, and they have a lot of public support or community support around them, but I think they still appreciate the fact that the offer was there”* [Police Participant B].

##### Areas for improvement

Some participants highlighted areas that could improve the potential effectiveness of the program for people bereaved by suspected suicide in the region. Bereaved participants described a desire for ongoing follow-up and support at later time points such as anniversaries and other important events: “*You get so much with so many people there with you in the initial stages, but it's down the track. I think you probably need it after the first year as well. it's always the first, the first birthday with kids and the first Christmas that's the hardest”* [Bereaved Participant D].

Bereaved participants also noted that it would be helpful to talk to someone who has lost someone to suicide who would understand their experience*: “I think something that I have tried to look up and get some access to is to just talk about it with other people that have possibly been through the same thing. I don't know if there's any group that you can go to. I did try to look up online but I couldn't find anything where – a bit like the Alcoholics Anonymous, so that sort of thing just so that you can get together with people who've experienced the same thing. For me, I think I'd benefit from that. I don't know if that even exists”* [Bereaved participant B].

Police and stakeholders noted that the effectiveness of the PCN model was conditional on the availability of support services in the community: “*Where the system will fall down is if the police are getting that filled out and sending it off, but the structures behind it aren't robust enough to be able to support the families*” [Police Participant B]. Concerns about the availability of continued funding for services and the implications this would have for bereaved participants was echoed by stakeholders who noted that further funding and central coordination was needed for the PCN model to be sustained in the community. One stakeholder noted: “*We're looking at a sector here that is very stretched and very understaffed, a lot of our mental health sector down in this area. [We need] the resourcing to draw everyone else together”* [Stakeholder Participant F].

### Discussion

This study sought to describe a novel community-led active postvention outreach intervention provided to individuals bereaved by suspected suicide. Results from the quantitative analysis provide key insights into the needs of individuals bereaved by suicide who received active outreach through the PCN model. Specifically, the present study found that between that for every suicide between 1 and 16 people were provided outreach from the PCN in the immediate aftermath of the suicide loss. The most common form of support provided to bereaved members of the community involved specialized postvention counseling, of which 92% attended at least one appointment. That between 1 and 16 individuals may benefit from proactive outreach may also have important implications for the planning and resources required to improve the reach of postvention services to those impacted by suicide in the community. This public health approach to postvention is warranted given previous research shows that 95% of bereaved individuals believe they need help accessing support, yet <50% of bereaved people actually receive it (20, 21, 23), despite multiple points of contact (e.g., from police, ambulance, funeral providers) in the immediate aftermath of a suicide (20, 21).

In the current study, participants who received support from the PCN model reported feeling a greater connection to their community, felt less isolation, and expressed that the intervention had the potential to prevent further deaths from occurring. Additionally, participants found that the practical support provided was highly beneficial (e.g., linking them to specialized postvention counseling, of which many bereaved participants did not know existed). Access to postvention counseling in the aftermath of a suicide loss has been identified as an essential need among individuals bereaved by suicide. Moreover, previous research conducted with people bereaved by suicide suggests that bereavement counseling and support groups is critical for recovery following a suicide loss (31). Despite this, previous studies indicate barriers such as lack of awareness of services, distance, cost and waitlist times can act as significant barriers to accessing support (23). Under the PCN model, barriers to help seeking are assessed during initial contact with bereaved individuals to facilitate better access to postvention services.

Previous studies report a positive association between a need for support and psychosocial complaints, trauma response, and complicated grief (20). In contrast, those who receive active postvention outreach, compared to passive postvention, show greater improvement in mental health and psychosocial outcomes (32, 33). For example, Gehrmann and colleagues found that individuals who received active postvention support reported significantly less suicidal ideation and social loneliness in the 12-months post-bereavement compared to those who sought support themselves (32). Moreover, being able to access support early after the loss also appears to be beneficial. Sanford and colleagues reported that those who recognize the need for help and obtain it earlier perceive greater benefit than those who access support later (33). Together, the findings from the present study provide further support outlining the benefits of an active-outreach approach to postvention for people in the community impacted by a suspected suicide.

In addition to supporting the needs of individuals bereaved by suicide, the present study found important secondary benefits for service providers in the community. It is well documented that police are frequently exposed to a range of occupational stressors including homicide, suicide and fatal accidents (34). These stressors place police at heightened risk for mental health morbidities, such as post-traumatic stress disorder and feelings of hopelessness, and suicidal ideation which can be exacerbated in working conditions where there is a perceived lack of support. In the present study, the PCN model was described by police as providing them with a sense of psychological comfort by allowing them to step away from a distressing situation knowing that individuals in distress would receive support within the next 24–72 h, should they need it. Additionally, police described these benefits to coincide with limited administrative burden, suggesting that the impact of the PCN model may be associated with wide reaching benefits within the community. These findings may underline the need of providing postvention training for first responders who are often exposed to suicide within their roles. A study by McDonnell and colleagues found that first responders trained in Postvention Assisting Those Bereaved by Suicide (PABBS) reported positive changes in knowledge, skills, and confidence in suicide bereavement (35).

Results from both the quantitative and qualitative findings provide further evidence that describe the practical needs of individuals during the immediate aftermath of a suspected suicide. Participants in our study received a wide range of postvention supports including education informing others about a suicide loss and referrals to suicide bereavement and specialist postvention counseling. Participants also received financial assistance, legal support, and transport assistance in addition to psychosocial support. It is noteworthy that many bereaved participants highlighted a need for peer support and long-term follow-up (e.g., at anniversaries and other important milestones) as areas of unmet needs. The potential benefits of peer support among people bereaved by suicide have been reported previously and include providing people with a chance to connect with others who have lived through similar or shared experiences (17). The unmet needs identified by participants in the present study provide important insights that could inform further service development of the PCN model in the PaRK region.

Taken together, the findings presented in the present study have important policy and practice implications for the design and delivery of postvention services more generally. Specifically, we show the benefits that may arise from establishing a collaborative pathway between police (or other first responders) and postvention services during the immediate aftermath of a suspected suicide. Importantly, the current study showed that the established referral pathway between police and the PCN was determined feasible with little administrative burden to first responders during a sudden death investigation. Second, the findings provide further support for a proactive outreach approach to postvention that provides practical support and in addition links individuals to specialist bereavement counseling. Lastly, individuals bereaved by suicide reported a need to connect with others who have lived experience of suicide themselves as well as opportunities for follow-up at different points during their bereavement journey. In response to this need, the PaRK suicide prevention response group has implemented a peer support program for individuals bereaved by suicide known as the Roses in the Ocean Peer CARE Companion program (36). This new peer support program has been implemented as a direct result of the current study, highlighting the benefits that can arise from program evaluation.

## Limitations

Several limitations should be noted when interpreting the study findings. In the absence of a control group or longitudinal follow-up, it is not possible to determine whether the current model leads to better grief outcomes among individuals who received active postvention support compared to those who receive passive postvention or no support. Moreover, further investigations are needed to assess whether the PCN model is a sustainable postvention approach in other communities which have fewer services or lack the capacity (e.g., the presence of long waitlist times for support services) that may prevent them from meeting the needs of individuals impacted by suicide in the community. In the present study, perceived effectiveness was investigated through interviews without statistical support to corroborate the qualitative findings. Future research that seeks to understand the specific characteristics of individuals who receive support from the PCN model has the potential to assist with service provision and planning. Lastly, it is possible that some individuals received postvention support in response to a death that had been misclassified as a suspected suicide. National data on suicide statistics in Australia suggest approximately 6% of suicides are recoded following a coronial investigation (37).

## Conclusion

The delivery of postvention support to people bereaved by suicide is a core component of suicide prevention strategies in Australia and internationally. The present study described the reach and perceived effectiveness of a novel community-led postvention intervention initiated by police following a suspected suicide. The results provide preliminary evidence supporting the perceived effectiveness of a proactive outreach approach that provides acute support to people bereaved by suspected suicide. The study highlights the importance of a public health approach to postvention that addresses the practical needs of individuals in addition to the psychological support to facilitate recovery in the immediate aftermath of a suicide loss.

## Data availability statement

The datasets presented in this article are not readily available because of the sensitivity of these data and the pending coronial investigation. Requests to access the datasets should be directed to nicole.hill@telethonkids.org.

## Ethics statement

The studies involving human participants were reviewed and approved by University of Western Australia Human Research Ethics Committee. The patients/participants provided their written informed consent to participate in this study.

## Author contributions

NH was responsible for the study design, data collection, data analysis, data interpretation, drafting the manuscript, and revising the manuscript. RW was responsible for data collection, data interpretation, and drafting the manuscript. PA was responsible for generating the figures. HB, KA, ST, AW, PS, YP, and AL were responsible for writing the manuscript interpreting the results and providing methodological support. All authors contributed to the article and approved the submitted version.
