# Supplementary figures and images for: Reach and perceived effectiveness of a community-led active outreach postvention intervention for people bereaved by suicide
Source: Front Public Health. 2022 Dec 22;10:1040323. doi: 10.3389/fpubh.2022.1040323 (PMC9815599; doi:10.3389/fpubh.2022.1040323)

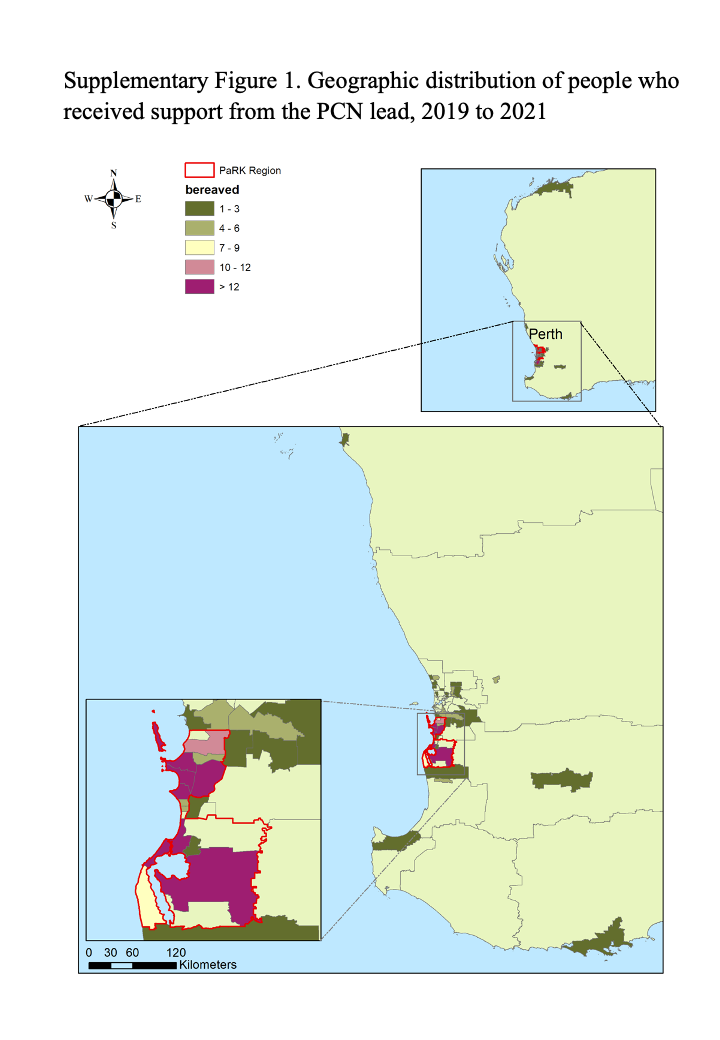

Supplement: Supplementary file 1 [file Image_1.TIFF]
